# Low prevalence of isoniazid preventive therapy uptake among HIV-infected patients attending tertiary health facility in Lagos, Southwest Nigeria

**DOI:** 10.11604/pamj.2021.39.123.28095

**Published:** 2021-06-13

**Authors:** Abdulwasiu Adeniyi Busari, Kazeem Adeola Oshikoya, Ifedolapo Adesola Adejumo, Olamide Ayinke Olanrewaju, Sikiru Olatunji Usman, Wasiu Adedeji Badru, Ibrahim Adekunle Oreagba, Sunday Oluwafemi Olayemi

**Affiliations:** 1 University of Lagos, College of Medicine, Lagos, Nigeria,; 2 Lagos State University, College of Medicine, Lagos, Nigeria

**Keywords:** Co-infection, human immunodeficiency virus, tuberculosis, isoniazid preventive therapy, policy, Nigeria, healthcare provider

## Abstract

**Introduction:**

the burden of HIV and tuberculosis co-infection is a global public health challenge. Despite the benefit of isoniazid preventive therapy (IPT) in reducing the rate of co-infection, the uptake is generally limited in developing countries. This study aimed to determine the prevalence of IPT use and the factors affecting the uptake among HIV-infected patients attending our Teaching Hospital.

**Methods:**

this cross-sectional survey involved 300 HIV-infected individuals attending the AIDS prevention initiatives in Nigeria clinic of the Lagos University Teaching Hospital. A self-designed and well-structured questionnaire was used to document the demographic data, patients’ exposure to tuberculosis, and IPT uptake. Clinical data of eligible patients were also extracted from their case notes. The main outcome measure was the prevalence of IPT use and non-use.

**Results:**

out of the respondents evaluated, (72.7%, n = 218) were females. Tuberculosis was the predominant comorbidity (15.7%, n = 47) and majority (53.0%, n = 159) had a CD4 count of < 500 cells/ml. Overall prevalence of IPT uptake was very low (7.1%, n = 18) among HIV-infected patients. Major factors affecting uptake were lack of awareness of benefit (44.4%, n = 8) and lack of fear of contracting tuberculosis (22.2%, n = 4). However, lack of awareness of IPT benefit was the only independent factor associated with poor IPT uptake (adjusted odds 1168.75, 95% confidence interval: 85.05-16060.33; p = 0.001).

**Conclusion:**

isoniazid preventive therapy uptake was found to be very low in this study. Increased awareness and policy implementation of IPT by the healthcare provider is necessary.

## Introduction

Human immunodeficiency virus (HIV) invades and destroys the immune system, thus increasing vulnerability to life-threatening opportunistic infections [1]. HIV is a global health challenge with associated morbidity and mortality. According to World Health Organization (WHO), HIV/AIDS has claimed more than 33 million lives over the past four decades [2]. Globally, tuberculosis is the most common opportunistic infection affecting people living with HIV infection and AIDS (PLWHA), a worrying trend for a treatable and curable illness [3]. The synergy between HIV and tuberculosis (TB) is strong. People living with HIV infection and AIDS are 16 to 27 times more likely to develop active tuberculosis than those without HIV, thus making HIV the strongest risk factor for acquiring TB [4]. Approximately one-third of deaths among HIV-positive patients were due to TB in 2018 [5]. Deaths among the HIV-infected populace caused by TB in 2015 were roughly 35%. Nonetheless, HIV-infected patients living in sub-Saharan Africa were about 71% out of an estimated 1.2 million new cases of HIV/TB co-infected people [6]. Tuberculosis/HIV co-infection accounted for about 208000 mortalities in 2019 [7]. The leading cause of death from HIV/ TB co-infection occurred in low- and middle-income countries [8]. Series of protocols to combat HIV/TB co-infection include HIV counseling and testing with the use of anti-retroviral and anti-tubercular drugs [9]. Nevertheless, morbidity and mortality associated with HIV/TB co-infection are still significantly high thus necessitating the implementation of Isoniazid preventive therapy (IPT) as part of the intervention for HIV/TB collaboration [10]. Nwokeukwu *et al*. in 2015 stated that IPT is the use of Isoniazid in HIV-positive individuals with latent TB infection [11].

Tuberculosis is a recognized risk to the achievable benefits of HIV scale-up care and treatment, thence making IPT a recommended strategy for reduction of mortality and morbidity in people living with HIV/AIDS [12,13]. World Health Organisation/Joint United Nations Program on HIV/AIDS(UNAIDS) issued a statement in 1998 that recommended IPT use for all HIV-infected patients living in the area with a high prevalence of latent TB with greater than 30% and those with documented latent TB infection or exposure to infectious TB cases regardless of their residence [12,13]. This was reinforced by the TB/HIV working group of the Stop TB Partnership in 2007 [14]. However, countries have been slow to adopt these recommendations and many limitations seem to be delaying effective nationwide implementation [15]. In 2007, only 30,000 representing 0.1% of people living with HIV worldwide had started IPT [16]. Despite the distinct benefits and various clinical trials showing the efficacy of IPT in ameliorating the huge burden of HIV/TB co-infection, the uptake of IPT remains low globally and specifically in sub-Saharan Africa. There appears to be a paucity of information on the prevalence of IPT in HIV-infected individuals in Nigeria and unless efforts are made to improve this, not much progress will be made to combat the overall devastating effect of the deadly duo. Data about factors affecting IPT uptake is rare. Therefore, this study aimed to assess the prevalence of uptake of IPT and associated factors affecting its use in HIV infected patients in Lagos University Teaching Hospital (LUTH).

## Methods

**Study design and location:** this study was conducted as a cross-sectional survey involving HIV-infected individuals attending Aids prevention initiatives in Nigeria (APIN) clinic at the Lagos University Teaching Hospital (LUTH). Participants were recruited over three months between 1^st^ August and 30^th^ October 2017. Aids prevention initiatives in Nigeria is a non-governmental and leading organization in the provision of prevention, care and treatment services to HIV-infected patients and other diseases of public health significance. There were 17,566 adult HIV-patients registered as of August 2017. Lagos University Teaching Hospital (LUTH) on the other hand is a tertiary health care center with an 800-bed capacity, situated in Surulere, Southwest Nigeria. It is a major referral center for the care of medical conditions in all specialties, including infectious diseases such as HIV/AIDS and tuberculosis.

**Study population:** HIV-infected patients were consecutively recruited from among those attending the APIN clinic using a convenient sampling method. Patients were enrolled into the study if ≥18 years old, HIV-infected, on ART for ≥3 months, and willing to give informed consent. Patients <18 years old, critically ill, pregnant, those already on treatment for TB, and non-consenting to the study were excluded. The sample size was calculated using Raosoft Online Sample Size Calculator with a power of 95% confidence level, response distribution set at 50%, 6% error margin, and population size of 17,566 patients which represented the total number of registered patients. The calculation yielded a sample size of 260. However, this was increased to 300 to make up for anticipated attrition and enable more patients to participate in the study.

**Study instrument:** data was obtained from eligible respondents using a well-structured self-designed questionnaire and case records of the individual patients. The questionnaire was interviewer-administered in which the trained interviewer asked questions from each eligible patient using a very simple language understood by the respondents. Data extracted included socio-demographic and clinical information such as age, gender, marital status, educational status, employment status, and income. Information on antiretroviral therapy (ART) regimen, duration of use, other co-prescribed medicines, patients´ exposure to tuberculosis, and IPT use were documented. Other clinical data of eligible patients were also extracted and they included the baseline laboratory parameters such as cluster of differentiation 4 (CD4) count, hemoglobin concentration, and viral load. The main outcome measure was the prevalence of IPT use and IPT non-use. The factors that affected the use of IPT were also predicted.

**Statistical analysis:** the data obtained were analyzed using Statistical Package for Social Sciences (SPSS®) version 22.0. (Chicago, Illinois, United States). Categorical variables were presented as percentages or proportions while continuous variables were presented as means + standard deviation and median + interquartile range. The demographic and clinical variables among IPT users and IPT non-users were compared. Multiple logistic regressions were used to determine the factor associated with IPT non-use. A p-value was regarded statistically significant if less than 0.05.

## Results

**Clinical and demographic characteristics of the respondents:** of the 300 participants enrolled in this study, (27.3%, n = 82) were males and (72.7%, n = 218) were females. The median age (years) + interquartile range was 41 years (36.00 - 47.00). Table 1 shows socio-demographic and clinical characteristics of the respondents. Majority (36.0%, n = 109) were in the age group between 35 - 44 years, only 2.3% were age > 65 years. Most patients were married (72.7%, n = 218), employed (77.7%, n = 233), had secondary education (47.7%, n = 143), no comorbidity (62.7%, n = 188), had CD4 count <500cell/mL (53.0%, n = 159) and had been on antiretroviral for less than 10 years (65.0%, n = 195). The prevalence of IPT uptake was found to be (7.1%, n = 18) overall among HIV-infected patients without TB co-infection. In Table 1, tuberculosis co-infection was the predominant comorbidity (15.7 %, n = 47), followed by hypertension (8.7%, n = 26), then hepatitis B (6.7%, n = 20) and hepatitis C (4.3%, n = 13). Others were diabetes (0.7%, n = 2) kidney disease (0.5%, n = 1) peptic ulcer (0.3%, n = 1) bronchial asthma (0.3%, n = 1) and sickle cell disease (0.2%, n = 1).

**Table 1:** socio-demographic and clinical characteristics of the respondents

Variable		Frequency	Percentage
Median age (interquartile range) years		41(36.0-47.0)	
**Age group**			
	<25	10	3.3
	25-34	63	21.0
	35-44	109	36.3
	45-54	86	28.7
	55-64	25	8.3
	> 65	7	2.3
**Gender**			
	Male	82	27.3
	Female	218	72.7
**Marital status**			
	Single	52	17.3
	Married	218	72.7
	Divorced	7	2.30
	Widowed	23	7.7
**Employment status**			
	Employed	233	77.7
	Unemployed	67	22.3
**Educational level**			
	None	4	1.3
	Primary	43	14.3
	Secondary	143	47.7
	Tertiary	110	36.7
**Comorbidity**			
	Yes	112	37.3
	Kidney disease	1	0.5
	Sickle cell	1	0.2
	Hypertension	26	8.7
	Hepatitis B	20	6.7
	Hepatitis C	13	4.3
	Diabetes	2	0.7
	Peptic Ulcer	1	0.3
	Asthma	1	0.3
	Tuberculosis	47	15.7
	No	188	62.7
**CD4 count (cells/ml)**			
	>500	141	47.0
	<500	159	53.0
**Duration on Anti-Retroviral therapy (year)**			
	>10	105	35.0
	<10	195	65.0
**Isoniazid preventive therapy use**			
	Yes	18	7.1
	No	235	92.9

**Figure 1:**
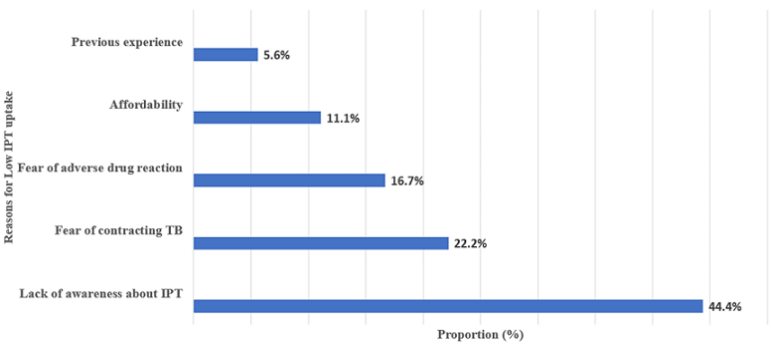
reasons for low IPT Uptake among HIV infected patients

**Analysis of factors affecting isoniazid preventive therapy uptake among the respondents:**Table 2 shows the comparison of demographic and clinical characteristics among IPT users and non-users. The proportion of IPT uptake was (7.1%, n= 18) while non-uptake was (92.9%, n= 236). There was no significant difference between age category > 60 years, gender, duration of antiretroviral use, presence of comorbidity, fear of adverse drug reaction, and CD4 count. However, IPT non-users were found to be significantly unaware of the benefit of IPT use and were less likely to fear contracting tuberculosis (p = 0.001). Figure 1 shows reasons for low IPT uptake among HIV-infected patients. The majority were due to a lack of awareness of IPT (44.4%, n = 8) followed by no fear of contracting tuberculosis (22.2%, n = 4). Others were fear of adverse drug reaction (16.7%, n = 3) and affordability (11.2%, n= 2).

**Table 2:** comparison of demographic and clinical characteristics among IPT users and non-users

Variable		IPT users N=18	IPT non-users N=236	P-value
**Age group** (years)				
	<60	17 (94.4)	223 (94.5)	1
	>60	1 (5.5)	13 (5.5)	
**Gender**				
	Male	7 (38.9)	61 (25.8)	0.269
	Female	11 (61.1)	175 (74.2)	
**Anti-retroviral therapy duration (years)**				
	>10	15 (83.3)	165 (69.9)	0.227
	<10	3 (16.7)	71 (30.1)	
**Presence of comorbidity**				
	Yes	2 (11.1)	38 (16.1)	0.747
	No	16 (88.9)	198 (83.9)	
**Awareness of isoniazid preventive therapy**				
	Yes	15 (83.3)	1 (0.9)	0.001
	No	3 (5.6)	235 (99.6)	
**Fear of contracting tuberculosis**				
	Yes	6 (33.3)	1 (0.4)	0.001
	No	12 (66.7)	235 (99.6)	
**Fear of adverse drug reaction**				
	Yes	1 (5.6)	1 (0.4)	0.137
	No	17 (94.4)	235 (99.6)	
**CD4 count (cells/ml)**				
	>500	3 (16.7)	100 (42.4)	0.044
	<500	15 (83.3)	136 (57.6)	

**Factors associated with low IPT uptake:**Table 3 shows univariate and multivariate analyses of the factors associated with low IPT uptake. After adjusting for other confounding variables such as age category > 60 years, gender, duration of antiretroviral use, presence of comorbidity, fear of adverse drug reaction, and CD4 count, only lack of awareness of IPT benefit was found to be independently associated with poor IPT uptake (adjusted odds 1168.75, 95% confidence interval: 85.05 - 16060.33; p = 0.001).

**Table 3:** univariable and multivariable analysis of factors associated with poor IPT uptake among HIV-infected patients

	Univariable analysis		Multivariable analysis	
	Unadjusted odds (95% confidence interval)	p-value	Adjusted odds (95% confidence interval	p-value
Age group (years)>60.096	1.009 (0.124- 8.182)	1	8.295 (0.69 - 99.752)	0
Gender (female)	1.826 (0.677 - 4.92)	0.227	-	-
Anti-retroviral therapy duration (years) >10	0.465 (0.130-1.656)	0.747	-	-
Presence of comorbidity	0.651 (0.144-2.950)		-	-
Awareness of isoniazid preventive therapy	1175 (115.174-11987.330)	0.001	1168.75 (85.05 - 16060.33)	0.001
Fear of contracting tuberculosis	117.5 (13.084-1055.185)	0.001	0.284 (0.012 - 6.775)	0.436
Fear of adverse drug reaction	13.824 (0.828 - 230.800)	0.137	4.42 (0.008 - 2600.600)	0.647
CD4 count (cells/ml) >500	0.272 (0.077 - 0.965)	0.044	0.328 (0.028 - 3.834)	0.3

## Discussion

Isoniazid prevention therapy (IPT) is an effective intervention in reducing the incidence of mortality and morbidity in PLWHA by preventing TB co-infection. The current recommended dose of IPT for adults is 300mg of isoniazid (INH) per day for 6 months, with 36 months conditionally recommended in areas of high TB prevalence and transmission [17]. A 2004 Cochrane review found that IPT reduced the risk of TB by 33% overall and by 64% when targeted at those with HIV with a positive tuberculin skin test. Furthermore, a retrospective cohort study in Addis Ababa, Ethiopia showed that IPT significantly reduced the incidence of TB by 96.3% compared to IPT non-exposed patients [18]. The uptake of IPT varies worldwide with uptake ranging from 2.4% to 73% in 2016 and the implementation is limited in developing countries falling short of the set global target of ≥ 90% where ironically the prevalence of HIV/TB is highest despite efforts and strategies to scale up the intervention [19-23]. We reported an IPT uptake of 7.1% in this study. This prevalence is extremely lower than the global set target of >90%. The low prevalence may be connected with poor awareness about IPT among the patients and the clinicians. Poor integration and implementation of IPT policy in our center may also be responsible. Although our findings are similar to those reported in some African countries where low uptake was reported [20-22]. Whilst, we reported one of the lowest uptakes, many other African and Asian countries documented higher prevalence in contrast to our study [21-23]. Apart from the study conducted in Malawi which reported IPT uptake of 6% [23] similar to our study despite the diversity in methodology designs, most countries with high HIV and TB burden like Nigeria reported higher prevalence invariance to our study. For instance, Timor Leste (18.7%), South Africa (26.8%), South India 33.0%, and Ethiopia (64.3%) of IPT uptake were reported [22,24].

Furthermore, IPT uptake in the Gambia, Rwanda, and Benin and were 89%, 89%, and 99% respectively. Although this was mainly reported in the pediatric population [24-26]. This may be explained by the integration of IPT into the programmatic delivery of healthcare in these countries. Interestingly, a study by van Griensven *et al*. in Cambodia showed a 100% uptake of IPT in a retrospective cohort study done between 2011-2013 using HIV/IPT program data [27]. While analyzing the factors affecting IPT uptake from the respondent´s perspective, we reported that lack of awareness and lack of fear of contracting TB was associated with low IPT uptake. Age, gender, duration of antiretroviral use, presence of comorbidity, and CD4 count status were not found to be associated with poor uptake. Our finding was similar to other studies where reasons for poor uptake were due to low awareness of IPT by patients and experience of health care workers as to knowledge and benefit of IPT. In one of the series, Jalo *et al*. 2020 while evaluating the uptake of Isoniazid preventive therapy for tuberculosis among HIV patients in Kano, Nigeria reported that those who lack awareness of IPT were less likely to uptake IPT (adjusted odds ratio (AOR) = 0.23, 95% CI = 0.08-0.68) similarly to our finding [28].

In our study, we documented that other reasons for poor uptake of IPT according to the patient´s perspective included fear of adverse drug reaction and affordability. Socio-demographic data were not statistically significant and did not affect the prevalence of IPT uptake in this study. Research by Adeniyi *et al*. in 2019 among pregnant adults with HIV showed a significant variation in the rate of IPT uptake when he compared geographical accessibility between rural and urban areas [29]. A similar trend was demonstrated in Bangkok, Thailand by Torneo *et al*. showing that a short distance from home to the nearest clinic improved adherence [30]. This study has some limitations, it was conducted as a single-center study. Also, factors affecting IPT uptake were assessed based on the patient´s perspective alone and not of health workers or policymakers. However, asides from these limitations, the study was able to identify that uptake of IPT was extremely low in this center. It implies a lot of work needs to be done in implementing this policy by prescribing the IPT to the patients. The implication of this finding is the need to improve IPT uptake by increasing awareness, counseling, removing barriers such as easier access to INH, improving capacity to diagnose TB, and screen, eligible patients. Also, measures to improve adherence to IPT and monitoring adverse drug reactions should be instituted.

## Conclusion

In conclusion, IPT uptake was found to be extremely low as it was initiated about 7 in every 100 eligible patients in this study. Lack of awareness of its benefit was an independent factor associated with poor uptake according to the patient´s perspective. Therefore, increase awareness and policy implementation of IPT by the healthcare provider is necessary.

### What is known about this topic

- HIV-infected patients are more susceptible to tuberculosis infection;
- Isoniazid Preventive Therapy (IPT) is shown to be effective in preventing TB/HIV co-infection;
- There is a varied prevalence of IPT uptake across the globe.

### What this study adds

- HIV-infected patients are more susceptible to tuberculosis infection;
- Isoniazid Preventive Therapy (IPT) is shown to be effective in preventing TB/HIV co-infection;
- There is a varied prevalence of IPT uptake across the globe.
